# Interpreting Posterior Relative Risk Estimates in Disease-Mapping Studies

**DOI:** 10.1289/ehp.6740

**Published:** 2004-04-15

**Authors:** Sylvia Richardson, Andrew Thomson, Nicky Best, Paul Elliott

**Affiliations:** Small Area Health Statistics Unit, Department of Epidemiology and Public Health, Imperial College Faculty of Medicine, Imperial College London, Norfolk Place, London, United Kingdom

**Keywords:** Bayesian hierarchical models, cancer mapping, environmental epidemiology, sensitivity, small-area studies, spatial smoothing, specificity

## Abstract

There is currently much interest in conducting spatial analyses of health outcomes at the small-area scale. This requires sophisticated statistical techniques, usually involving Bayesian models, to smooth the underlying risk estimates because the data are typically sparse. However, questions have been raised about the performance of these models for recovering the “true” risk surface, about the influence of the prior structure specified, and about the amount of smoothing of the risks that is actually performed. We describe a comprehensive simulation study designed to address these questions. Our results show that Bayesian disease-mapping models are essentially conservative, with high specificity even in situations with very sparse data but low sensitivity if the raised-risk areas have only a moderate (< 2-fold) excess or are not based on substantial expected counts (> 50 per area). Semiparametric spatial mixture models typically produce less smoothing than their conditional autoregressive counterpart when there is sufficient information in the data (moderate-size expected count and/or high true excess risk). Sensitivity may be improved by exploiting the whole posterior distribution to try to detect true raised-risk areas rather than just reporting and mapping the mean posterior relative risk. For the widely used conditional autoregressive model, we show that a decision rule based on computing the probability that the relative risk is above 1 with a cutoff between 70 and 80% gives a specific rule with reasonable sensitivity for a range of scenarios having moderate expected counts (~ 20) and excess risks (~1.5- to 2-fold). Larger (3-fold) excess risks are detected almost certainly using this rule, even when based on small expected counts, although the mean of the posterior distribution is typically smoothed to about half the true value.

Spatial analyses of health outcomes have long been recognized in the epidemiologic literature as playing a specific and important role in description and analysis. In particular, they can highlight sources of heterogeneity underlying spatial patterns in the health outcomes and consequently are able to suggest important public health determinants or etiologic clues. A good example of geographic epidemiology is the seminal monograph by Doll (1980), which described some of the first hypotheses concerning the influence of environment and lifestyle characteristics on cancer mortality and discussed how these arose from studying the geographic distribution of various cancers. These early studies were usually performed on a large geographic scale, using mostly international or regional comparisons.

Recently, the availability of local geographically indexed health and population data, together with advances in computing and geographic information systems, has encouraged the analysis of health data on a small geographic scale (Elliott et al. 2000). The motivation is the increased interpretability of small-scale studies, as they are in principle less susceptible to the component of ecologic bias created by the within-area heterogeneity of exposure or other determinants. They are also better able to detect highly localized effects such as those related to industrial pollution in the vicinity. Conversely, small-scale studies require more sophisticated statistical analysis techniques than, for example, an analysis between countries, because the data are typically sparse with low (even zero) counts of events in many of the small areas. Further, frequently there is evidence of overdispersion of the counts with respect to the Poisson model as well as spatial patterns indicating some dependence between the counts in neighboring areas.

Faced with these nonstandard characteristics, statistical models have been developed to address these issues and make best use of small-area health data. In connection with generic developments in a flexible modeling strategy using the paradigm of Bayesian hierarchical models, hierarchical disease-mapping models based on conditional autoregressions (CAR) were proposed in the 1990s through the work of Besag et al. (1991), Clayton and Bernardinelli (1992), and Clayton et al. (1993). These CAR models are now commonly used both by statisticians and epidemiologists, and their implementation is facilitated by existing software such as WinBUGS (Spiegelhalter et al. 2002). Alternative semiparametric formulations to CAR have also been proposed recently (Denison and Holmes 2001; Green and Richardson 2002; Knorr-Held and Rasser 2000) to model more heterogeneous risk surfaces and particularly to allow for potential discontinuities in the risk. The main characteristic of all these models is to provide some shrinkage and spatial smoothing of the raw relative risk estimates that otherwise would be computed separately in each area. Such shrinkage gives a more stable estimate of the pattern of underlying risk of disease than that provided by the raw estimates. The pattern of the raw risks, strongly influenced by the size of the population at risk, leads to a noisy and blurred picture of the true unobserved risks.

Within the disease-mapping paradigm, questions have been raised about the performance of these models in recovering the true risk surface, the influence of the prior structure specified, and the amount of smoothing of the risks actually performed by these models. In other words, it is important to understand thoroughly the sensitivity (ability to detect true patterns of heterogeneity of risk) and the specificity (ability to discard false patterns created by noise) of Bayesian disease-mapping models. This is the focus of this article. This understanding is crucial for interpretation of any specific disease pattern derived through the use of such models. Such a calibration study cannot be performed on real data because it relies on knowing the true underlying pattern of risk. We have thus conducted an extensive simulation study where the generated data patterns are close to those found in typical disease-mapping studies. We report here the main conclusions that can be drawn.

Let us stress that we are not placing ourselves in the context of cluster detection methods based on so-called point data, that is, data where the precise geographic location of all the cases (and controls) is known. These methods, which have been reviewed in a number of monographs or special issues (e.g., Alexander and Boyle 1996) are typically used on a localized scale, mostly to study the spatial distribution of cases around a point source or different patterns of randomness or clustering of the cases in relation to those of controls. Here we are concerned with methods for describing the overall spatial pattern of cases aggregated over small areas and the interpretation of the residual variations once the Poisson noise has been smoothed out by the disease-mapping models. Several simulation exercises to study different aspects of the performance of disease-mapping models have been reported recently. For example, Lawson et al. (2000) compared a number of models that could be used for disease mapping by goodness of fit criteria that included correlation between the simulated map and the smoothed map and a Bayesian information criterion. They concluded that the version of the CAR model proposed by Besag et al. (1991) [Besag, Yorke, and Mollié (BYM) model] was the most robust model among those with spatial structure. We also consider the BYM model here and compare its performance with two models not considered by Lawson et al. (2000): a version of the BYM model that is more robust to outliers and hence may be better able to detect abrupt changes in the spatial pattern of risk, and a spatial mixture model proposed by Green and Richardson (2002) (see the section on Bayesian disease-mapping models for details). Our study also specifically addresses the smoothing of high-risk areas and further use of the posterior distribution of the relative risks for detecting areas with excess risk—issues not considered by Lawson et al. (2000). Jarup et al. (2002) reported the results of a small simulation study similar to ours; we chose the same study area and expected counts to carry out our comprehensive exercise.

In the next section we describe the simulation setup and the Bayesian disease-mapping methods to be compared. We then discuss the interpretation of the means of the posterior distribution of the relative risk estimates typically reported in disease-mapping studies and displayed as summary maps, and we illustrate quantitatively how much smoothing is performed. We then discuss how information on the whole posterior distribution of the relative risks can be better exploited to discriminate between areas displaying higher risk and areas with relative risk close to background level. We conclude with a short discussion that emphasizes the importance of interpreting any results from a disease-mapping exercise in the context of the size of expected counts and the potential spatial structure of the risks.

## Materials and Methods

The basic setup of disease-mapping studies is as follows: The number of cases of a particular disease *Y_i_* occurring in area *A_i_* is recorded, where the set of areas {*A_i_*},*i* = 1, 2,…,*n* represents a partition of the region under study. For each area *A_i_*, the expected number of cases *E_i_* is also computed using reference rates for the disease incidence (or mortality) and the sociodemographic strata (with respect to age, sex, and perhaps socioeconomic characteristics) where census data are available.

The distribution of the counts *Y_i_* is typically assumed to come from a Poisson distribution, as the diseases usually considered in such studies are rare and this distribution gives a good approximation to the underlying binomial distribution that would hold for each risk stratum. The local variability of the counts is thus modeled as follows:


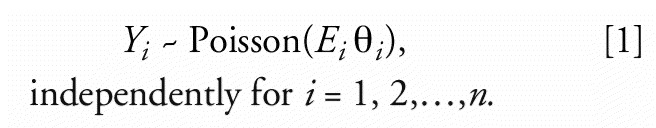


The parameter of interest is θ*_i_*, the relative risk that quantifies whether the area *i* has a higher (θ*_i_* > 1) or lower (θ*_i_* < 1) occurrence of cases than that expected from the reference rates. It is this parameter that we are trying to estimate to quantify the heterogeneity of the risk and to highlight unusual patterns of risks.

### Data Generation

The spatial structure used throughout the simulations is that of the 532 wards in the county of Yorkshire, England. Wards are administrative areas in the United Kingdom, with a total population of approximately 5,000 on average. We base our expected counts *E_i_* on those calculated by Jarup et al. 2002 for prostate cancer in males 45–64 years of age over the period from 1975 to 1991. We then simulate three spatial patterns of increased risks. For each pattern, we examine three magnitudes for the elevated risks. We also examine how the inference is changed if the expected counts are multiplicatively increased by a scale factor (SF) varying from 2 to 10.

Three spatial patterns for areas of elevated risk were chosen. The choice of patterns was intended to span a spectrum ranging from a scenario with single isolated areas with elevated risks (the hardest test case for any smoothing method) to a scenario with a number of larger clusters of several contiguous areas with elevated risks (a situation with a substantial amount of heterogeneity). In all cases the elevated areas were selected in turn at random from the set of areas with the required expected counts. In the Simu 1 and Simu 3 cases, once an area was selected, a buffer of neighboring areas with background risk (excluded thereafter from the random selection) was placed around it to produce the required pattern of isolated high-risk clusters. The three generated patterns were defined as follows:

- Simu 1: five isolated single wards with expected counts ranging from 0.8 to 7.3 corresponding, respectively, to the 10th, 25th, 50th, 75th, and 90th percentiles of the distribution of the expected counts
- Simu 2: a group of contiguous wards representing 1% of the total expected counts. In effect, this chosen 1% cluster grouped four wards with fairly comparable expected counts ranging from 3.6 to 7.0, giving an average expected count per ward of 5.4 over the four wards
- Simu 3: a situation with high heterogeneity comprising 20 such 1% clusters that are nonoverlapping.

Note that for Simu 3, the twenty 1% clusters each have a total expected count close to 17 but a large disparity in terms of numbers of constitute areas: 10 clusters had 2 or 3 areas, whereas 8 clusters had more than 8 areas, up to a maximum of 18 areas. Correspondingly, the expected counts in each of the wards in the clusters ranged from 0.3 for some wards in the 18-area cluster to 12 for the cluster with 2 areas. Simu 3 thus corresponds to a realistic situation of heterogeneity of risk where both small clusters with high expected counts, for example, typically a populated area, and large clusters each with small expected counts, for example, in rural areas, are present. This high degree of heterogeneity has to be considered when interpreting the results for the Simu 3 case where an average over all the 20 clusters is presented. Note also that contrary to the Simu 2 case, about half the background areas in Simu 3 have a neighbor that belongs to one of the 20 clusters. In each case, apart from the elevated risk areas described above, all other areas are called background areas.

For each spatial pattern in Simu 1 and Simu 2, counts *Y_i_* were generated as follows: Counts in all background areas were generated from a Poisson distribution with mean *E_i_*. For all the other areas, an elevated relative risk with magnitude θ*_i_* > 1 was used and counts were simulated as Poisson variables with mean θ*_i_E_i_* . The simulation was repeated for three values of θ*_i_* (1.5, 2, and 3) and for different SFs that multiply the expected counts *E_i_* for all areas. Thus, results reported, for example, for an area with *E* = 1.92, θ = 2, and SF = 4, correspond to counts generated from a Poisson with mean 15.36 (2 × 4 × 1.92).

For Simu 3 a slightly different procedure for generating the cases was used to ensure that Σ *Y_i_* = Σ *E_i_* (Appendix A). Note that for Simu 1 and Simu 2, the simulation procedure meant only that Σ*Y_i_* ≈ Σ*E_i_*. This corresponds, for instance, to an epidemiologic situation where expected counts *E_i_* are calculated based on an external reference rate. However, Simu 3 uses internal reference rates because otherwise Σ*Y_i_* would have been much larger than *E_i_* , which could distort the overall risk estimates. The multinomial procedure used in Simu 3 and detailed in Appendix A implies that, in effect, the multiplicative contrast between areas of elevated risk and background areas is still 3, 2, and 1.5, respectively, but the corresponding relative risks in each area (denoted * θ *_i_* ) relative to the internal (i.e., study region average) reference rates are now 2.1, 1.65, and 1.35 for the elevated areas and 0.7, 0.82, and 0.9 for the background areas.

To allow for sampling variability, each simulation case was replicated 100 times. The results presented are averaged over these 100 replications. A total of 36 simulation scenarios were investigated, corresponding to three spatial patterns (Simu 1, 2, and 3) × three different magnitudes of elevated risk (θ = 3, 2, and 1.5) × 4 SFs for the expected counts *E_i_* (SF = 1, 2, 4, and 10).

### Bayesian Disease-Mapping Models

Bayesian disease-mapping models treat the relative risks {θ*_i_* } as random variables and specify a distribution for them. This part of the model is crucial, as the distributional assumptions thus made allow borrowing of information across the areas. The distribution specified is referred to as the second hierarchical level of the model to distinguish it from the first-level distribution specified in equation 1 that pertains to the random sampling variability of the observed counts about their local mean. It is at this second level that the spatial dependence between the relative risks is introduced. This spatial dependence is represented by means of a prescribed neighborhood graph that defines the set of neighbors (denoted by ∂*_i_*) for each area *i*.

The most commonly used parametric model at the second level of the hierarchy is the CAR model. This specifies the distribution of the log relative risks *v_i_* = log(θ*_i_* ) by


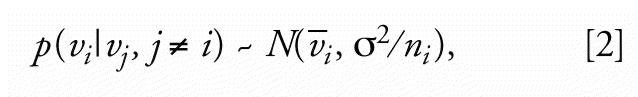


where σ^2^ is an unknown variance parameter, and &*vmacr;_i_* *=* Σ*_j_*_∈∂_*_i_v_j_/n_i_* , where *n_i_* is the number of neighbors of area *i*. Thus, essentially the log relative risk in one area is influenced by the average log relative risk of its neighbors, with variability characterized by a conditional variance σ^2^*/n_i_*

This CAR model makes a strong spatial assumption and has only one free parameter linked to the conditional variance σ ^2^. To increase flexibility, Besag et al. (1991) recommend modeling log (θ*_i_*) as the sum of a CAR process and an unstructured exchangeable component δ*_i_* *~ N*(0,τ^2^), *i* = 1, …,*n* independently:


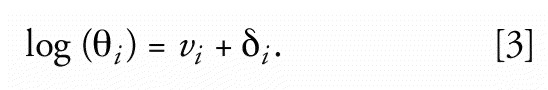


This is the BYM model introduced by Besag et al. (1991) that we referred to earlier. We use this model as a benchmark, as its use in disease-mapping studies has been widespread since 1991.

The Gaussian distribution used in the CAR specification above induces a high level of smoothness. In the same 1991 article, Besag et al. (1991) discussed an alternative specification using the heavier-tailed, double-exponential distribution rather than the Gaussian distribution in Equation 2. In effect, this is similar to performing a median-based local smoothing (or L1 norm) rather than a mean-based smoothing, thus allowing more abrupt changes in the geographical pattern of risk. We will refer to this model as L1-BYM.

With any such parametric specification, the amount of smoothing performed (e.g., controlled by the parameters σ^2^ and τ^2^) is affected globally by all the areas and is not adaptive. Concerns that such parametric models could oversmooth have led several authors to develop semiparametric spatial models that replace the continuously varying spatial distribution for {θ*_i_*} by discrete allocation or partition models. Such models allow discontinuities in the risk surface and make fewer distributional assumptions. Partition models that allow a variable number of clusters have been proposed by Denison and Holmes (2001) and Knorr-Held and Rasser (2000).

In this article we investigate the performance of a related spatial mixture model recently proposed by Green and Richardson (2002) that we refer to as MIX. This model leads to good estimation of the relative risks compared with the BYM model for a variety of cases of discontinuities of the risk surface. The idea underlying the MIX model is to replace a continuous model for θ*_i_* by a mixture model that uses a variable number of risk classes and a spatially correlated allocation model to distribute each area to a class. By averaging over a large number of possible configurations, the marginal distribution of the relative risk is nevertheless smooth. To be precise, it is assumed that θ*_i_* *=* θ*_Zi_* , where *Z_i_*, *i* = 1, 2,…,*n* are allocation variables taking values in 1, 2,…,*k* and θ*_j_, j* = 1,2,…,*k* are the values of the relative risks that characterize the *k* different components or risk classes. To have maximum flexibility, the number of components *k* of the mixture is treated as unknown. Given *k*, the allocations *Z_i_* follow a spatially correlated process, the Potts model, which has been used in image processing and other spatial applications and involves a positive interaction parameter ψ (similar to an autocorrelation parameter) that influences the degree of spatial dependence of the allocations. Specifically, the allocation of an area to a risk component will be favored probabilistically by the number of neighbors currently attributed to that component scaled multiplicatively by ψ. In this way the prior knowledge that areas close by tend to have similar risks can be reflected through the allocation structure. The interaction parameter ψ is treated as unknown and jointly estimated with the number of components and their associated risk. The MIX model can adapt to various patterns of risk and model discontinuities by creating a new risk class if there is sufficient information in the data to warrant this. Further details on the specification of the model are given in Green and Richardson (2002). Thus, in the comparison described later, we have implemented one reference model BYM and two alternative models, the parametric L1-BYM and the semiparametric MIX model.

### Implementation

Bayesian inference is based on the joint posterior distribution of all parameters given the data. In our case this joint distribution is mathematically intractable and is simulated using the framework of Markov chain Monte Carlo techniques now commonly used in Bayesian analyses (Gilks et al. 1996). All parameters involved in the models described above, for example, the variances σ^2^ or τ^2^ or the interaction parameter ψ, are given prior distributions at a third level of the hierarchy. Implementation of the BYM and L1-BYM was carried out using the free software WinBUGS (Spiegelhalter et al. 2002). Implementation of the MIX model was carried out using a purpose-built Fortran code.

## Results

### How Smooth Are the Posterior Means?

The results of a Bayesian disease-mapping analysis are typically presented in the form of a map displaying a point estimate (usually the mean or median of the posterior distribution) of the relative risk for each area. To interpret such maps, one needs to understand the extent to which the statistical model is able to smooth the risk estimates to eliminate random noise while at the same time avoiding oversmoothing that might flatten any true variations in risk. To address this issue, we consider the two aspects separately: *a*) do the Bayesian methods provide adequate smoothing of the background rates, and *b*) to what extent is the posterior mean estimate different from the background risk in the small number of areas simulated with a true elevated risk?

**Figure 1:**
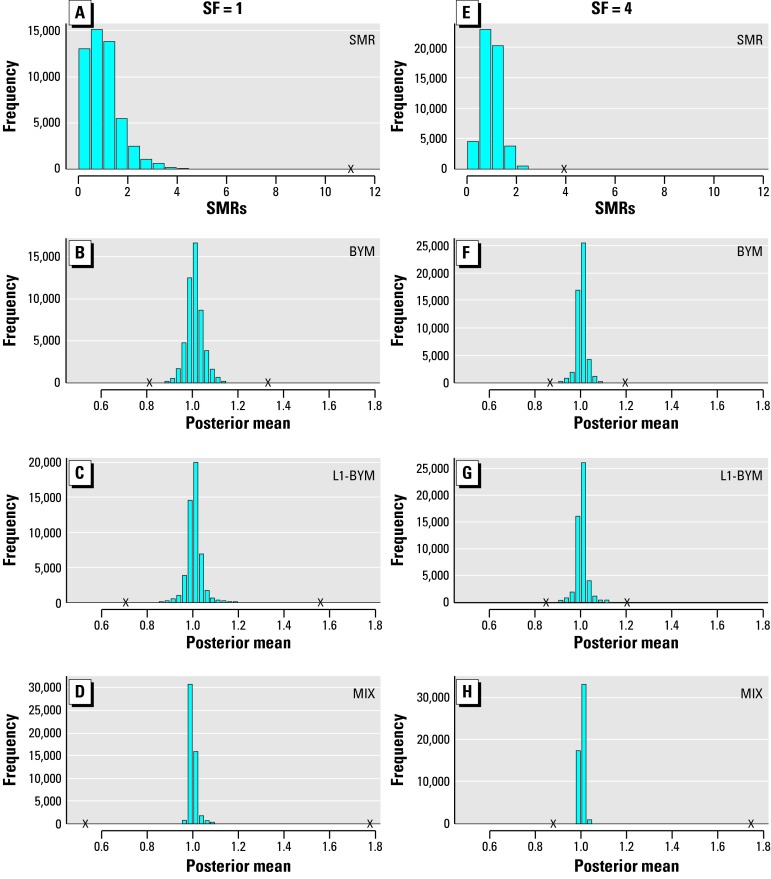
Histograms of the raw SMRs (*A,E* ) and posterior means of the relative risks (*B–H*) for all the background areas of Simu 2 derived by each of the three models. Note that the crosses on the *x-*axes indicate the minimum and maximum values obtained. SF indicates the scale factor used for the expected values.

In all the cases simulated, we found substantial shrinkage of the relative risk estimates for the background rates. This is well illustrated in Figure 1, which displays raw and smooth estimates for all the background areas of Simu 2 and an SF of 1 or 4. Note that when SF = 1, the histogram of the raw standardized mortality or morbidity ratio (SMR) estimates is very dispersed (Figure 1A), with a range of 0–11, and shows a skewed distribution. Clearly, mapping the raw SMRs would present a misleading picture of the risk pattern, whereas any of the three Bayesian models give posterior mean relative risk estimates for the background areas that are well centered on 1 (Figure 1B–D), with just a few areas having estimates outside the 0.9–1.1 range. When the expected counts are higher (SF = 4), the histogram of the raw SMRs is less spread but still substantially overdispersed, whereas those corresponding to the three models are even more concentrated on 1 than when SF = 1 (Figure 1F–H). Thus the false patterns created by the Poisson noise are adequately smoothed out by all the disease-mapping models.

Details of the performance of the BYM model in estimating the relative risk of the high-risk areas are presented in Table 1, with findings for L1-BYM and MIX shown in Tables 2 and 3, respectively. Overall, for the BYM model, a great deal of smoothing of the relative risks is apparent. For the isolated areas in Simu 1, one can see that relative risks of 1.5 in any single area are smoothed away, even in the most favorable case of an area with expected counts of 70 (90% area SF = 10). When the simulated relative risk is 2, the posterior mean risk estimate is above 1.2 only when the expected count is around 50 or more (e.g., 75% area with SF = 10). Relative risks of 3 are smoothed to about half their values when the expected counts are around 10 (e.g., 25% area with SF = 10 or 75% area with SF = 2). Comparison of Simu 2 with Simu 1 (75% area) shows that having a cluster of high-risk areas rather than a single area with elevated risk slightly decreases the amount of smoothing for the same average expected count. Again, this is apparent in the many-cluster situation of Simu 3, where even though the true θ^*^_i_ are smaller, the relative risk estimates are higher than those for Simu 2.

Overall, the performance of the L1-BYM model (Table 2) is similar to that of the BYM model. However, as expected, the L1-BYM model effects a little less smoothing in cases of large expected counts or high relative risk estimates. For Simu 3 the estimates are nearly identical to those of the BYM model. Thus, simply changing the distributional assumptions in the autoregressive specifications results in only a small modification in the estimates.

The results for the MIX model given in Table 3 show a different pattern than those for the BYM or L1-BYM. For Simu 1 and an elevated relative risk of 1.5, strong smoothing toward 1 is apparent as for BYM. However, for Simu 2, posterior mean relative risks become higher than 1.2 for the largest SF. At the other end of the spectrum, relative risks of 3 are well estimated with posterior means above 2.5 as soon as the expected count is above 10 either for single areas (e.g., 50% area with SF = 4) or for the 1% clustered areas with SF = 2. These results are in accordance with the nature of the MIX model. When there is sufficient evidence in the data to create a group of areas with higher risk, the posterior mean risks for the areas in this group are well estimated and close to the simulated values. Otherwise, all areas are allocated to the background category and smoothed toward 1.

**Figure 2:**
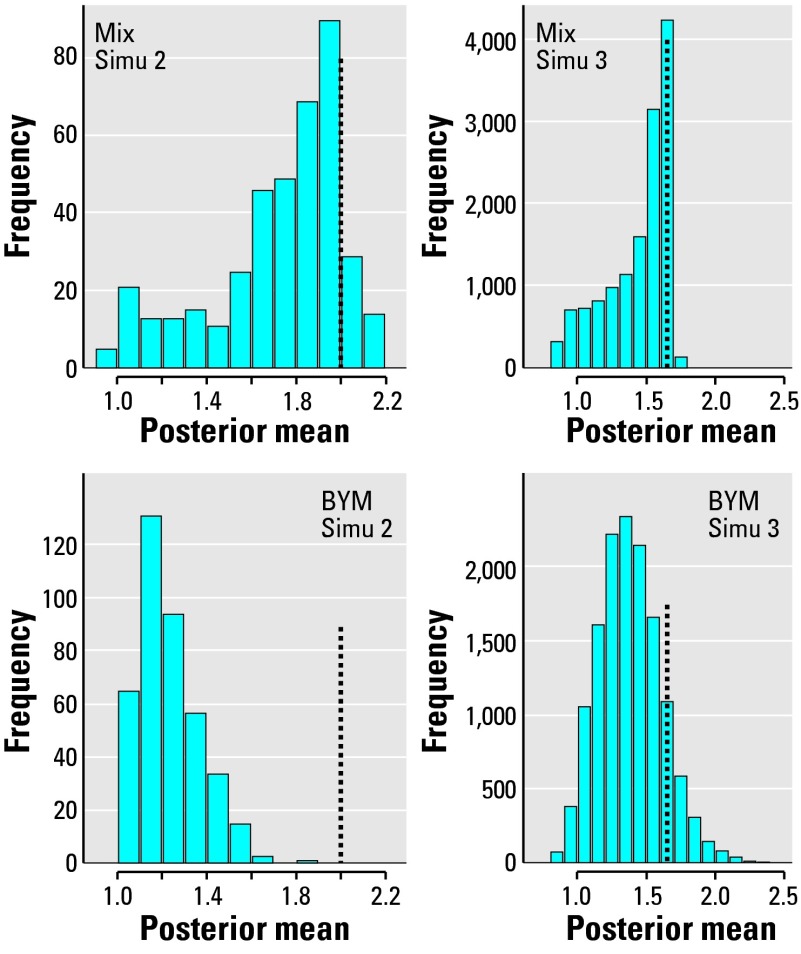
Histograms comparing the distribution of the posterior means of the relative risks estimated by the BYM or MIX models for the high-risk areas of Simu 2 or Simu 3 using a scale factor of 4 for the expected values and a true relative risk (marked by the vertical line on each plot) of θ = 2 (Simu 2) or θ *1=1.65 (Simu 3).

Having many heterogeneous clusters as in Simu 3 does not improve the MIX performance as much as that of BYM. Because of the more diffuse nature of some of the clusters, more areas in the background are randomly included in the group of areas with higher risk. Thus, the MIX model still has a mode close to the true relative risk, but the histogram of the mean posterior risks for all the high-risk areas has a longer left-hand tail than in the Simu 2 scenario (Figure 2).

**Figure 3:**
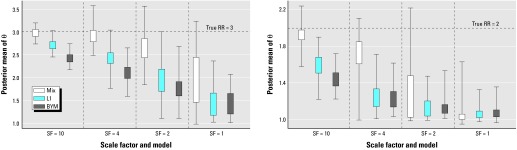
Box plots of the posterior means of the relative risks estimated by the three models for the high-risk areas of Simu 2 as a function of the scaling factor.

The difference in performance of the three models is further illustrated in Figure 3, which displays, for the three models, box plots of the posterior mean estimates of the relative risk in the raised-risk areas over the 100 replicates for Simu 2 with true relative risks of 3 and 2. When the true relative risk is 3, the MIX model is clearly performing better than the other two models, whereas for a relative risk of 2 and the lowest SF, the MIX model is the model that produces the most smoothing.

### Interpreting the Posterior Distribution of the Risk

**Figure 4:**
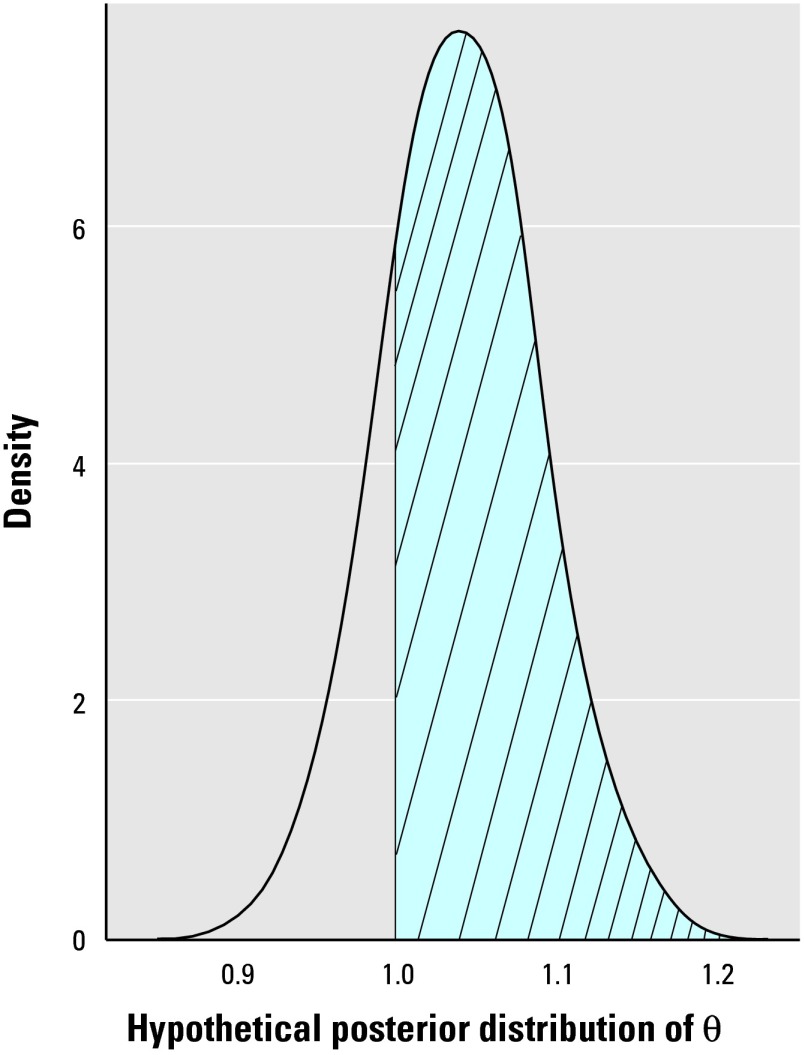
Posterior distribution of a relative risk θ, with shaded area indicating Prob(θ > 1).

Mapping the posterior mean relative risk as discussed previously does not make full use of the output of the Bayesian analysis that provides, for each area, samples from the whole posterior distribution of the relative risk. Mapping the probability that a relative risk is greater than a specified threshold of interest has been proposed by several authors [e.g., Clayton and Bernardinelli (1992)]. We carry this further and investigate the performance of decision rules for classifying an area *A_i_* as having an increased risk based on how much of the posterior distribution of θ*_i_* exceeds a reference threshold. Figure 4 presents an example of the posterior distribution of the relative risk for such an area. The shaded proportion corresponds to the posterior probability that θ > 1. To be precise, to classify any area as having an elevated risk, we define the decision rule *D*(*c, R*_0_), which depends on a cutoff probability *c* and a reference threshold *R*_0_ such that area *A_i_* is classified as having an elevated risk according to *D*(*c, R*_0_) ↔ Prob(θ*_i_* > *R*_0_) > *c*. The appropriate rules to investigate will depend on the shape of the posterior distribution of θ*_i_* for the elevated areas. We first discuss rules adapted to the autoregressive BYM and L1-BYM models. For these two models we have seen that, in general, the mean of the posterior distribution of θ*_i_* in the raised-risk areas is greater than 1 but rarely above 1.5 in many of the scenarios investigated. Thus, it seems sensible to take *R*_0_ = 1 as a reference threshold. We would also expect the bulk of the posterior distribution to be shifted above 1 for these areas, suggesting that cutoff probabilities well above 0.5 are indicated. In the first instance, we choose *c* = 0.8. Thus, for the BYM and L1-BYM models, we report results corresponding to the decision rule *D*(0.8, 1). See Appendix B for a detailed justification of this choice of value of *c* and the performance of different decision rules.

In contrast, we have seen that the mean of the posterior distribution of θ*_i_* for raised-risk areas for the MIX model is closer to the true value for many scenarios, and there is clear indication that the upper tail of this distribution can be well above 1. Furthermore, the spread of this distribution is less than the corresponding one for the BYM or L1-BYM models, as noted by Green and Richardson (2002). The choice of threshold is thus more crucial for this model, making it harder to find an appropriate decision rule. After some exploratory analyses of the simple clusters in Simu 1 and Simu 2, we found that a suitable decision rule for the MIX model in these two scenarios is to choose *R*_0_ = 1.5. For such a high threshold, one would expect that it is enough for a small fraction (e.g., 5 or 10%) of the posterior distribution of θ*_i_* to be above 1.5 to indicate that an area has elevated risk. Thus, for the MIX model we report results corresponding to the decision rule *D*(0.05, 1.5).

Two types of errors are associated with any decision rule: *a*) a false-positive result, that is, declaring an area as having elevated risk when in fact its underlying true rate equals the background level (an error also traditionally referred to as type I error or lack of specificity); and *b*) a false-negative result, that is, declaring an area to be in the background when in fact its underlying rate is elevated (an error also referred to as type II error or lack of sensitivity). In epidemiology, performances are discussed either by reporting these error rates or their complementary quantities that measure the success rates of the decision rule. The two goals of disease mapping can be summarized as follows: not to overinterpret excesses arising by chance, that is, to minimize the false-positive rate but to detect patterns of true heterogeneity, that is, to maximize the sensitivity. We thus choose to report these two easily interpretable quantities. To be precise, for any decision rule *D(c, R*_0_*)*, we compute

- the false-positive rate (or 1 – specificity), that is, the proportion of background areas falsely declared elevated by the decision rule *D(c, R*_0_*)*
- the sensitivity (or 1 – false-negative rate), that is, the proportion of areas generated with elevated rates correctly declared elevated by the decision rule *D(c, R*_0_*)*.

It is clear that there must be a compromise between these two goals: a stricter rule (i.e., one with a higher value of *c* or *R*_0_ or both) reduces the false-positive rate but also decreases the sensitivity and thus increases the false-negative rate. Thus, to judge the performance of any decision rule, one has to consider both types of errors, not necessarily equally weighted. See Appendix B for an illustration of the implication of different weighting on the overall performance of the decision rule.

Table 4 summarizes the probabilities of false-positive results for the three models. For BYM and L1-BYM, the probabilities stay below 10% with no discernible pattern for Simu 1 and Simu 2. The error rates are clearly smaller and around 3% for Simu 3. In this scenario, the background relative risk is shifted below 1, so a decision rule with *R*_0_ = 1 is, in effect, a more stringent rule than in the case of Simu 1 and Simu 2 where the background relative risks are close to 1. For the MIX model, the false-positive rates are quite low for Simu 1 and Simu 2 and stay mostly below 3%. However, as shown in the last line of Table 4, these rates have greatly increased for the Simu 3 scenario, indicating that the decision rule *D*(0.05, 1.5) is no longer appropriate in this heterogeneous context. The heterogeneity creates a lot of uncertainty, with some background areas being grouped with nearby high-risk areas; consequently, the rule *D*(0.05, 1.5) is not stringent (specific) enough. Thus, we have investigated a series of rules *D(c*, 1.5) for *c* = 0.1–0.4 for the MIX model in the Simu 3 scenario. As *c* increases, the probability of false positive decreases; for *D*(0.4, 1.5), the probability is, on average, around 3% and always below 7% (Table 5).

Concerning the detection of truly increased relative risks and sensitivity, we first discuss the results for the BYM and L1-BYM models. As expected from the posterior means shown in Tables 1 or 2, the ability to detect true increased risk areas is limited when the increase is only of the order of 1.5. If one takes as a guideline the cases where the detection of true positive is 50% or more, Tables 6 and 7 show that this sensitivity is reached for an expected count of around 50 in the case of a single isolated area and around 20 for the 1% cluster scenario. This shows that for rare diseases and small areas, there is little chance of detecting increased risks of around 1.5 while adequately controlling the false-positive rate.

True relative risks of 2 are detected with at least 75% probability when expected counts are between 10 and 20 per area, depending on the spatial structure of the risk surface, whereas true relative risks of 3 are detected almost certainly when expected counts per area are 5 or more. There is no clear pattern of difference between the results for BYM and L1-BYM; overall, the sensitivity is similar. For Simu 3 we see that the sensitivity is lower than for the other simulation scenarios with equivalent expected counts (as were the rates of false positive in Table 4), in line with the true relative risks being closer to 1 than for Simu 1 and Simu 2. Hence, the decision rule *D*(0.8, 1) is more specific but less sensitive in this scenario. In situations comprising a large degree of heterogeneity akin to Simu 3, it thus might be advantageous to consider alternative rules, even if the rate of false positive is less well controlled. For example, in the case of a true relative risk (θ) = 1.65 and SF = 4, the use of rule *D*(0.7, 1) for the BYM model leads to a higher probability of false positive (6% compared with the 3% shown in Table 4). However, the corresponding gain in sensitivity is more than 10%, with the probability of detecting a true positive increasing to 82% compared with 71% when using the rule *D*(0.8, 1) (Table 5). Nevertheless, even with this relaxed and more sensitive rule, the chance of detecting a true relative risk as small as 1.3 is only around 50% if the SF is 4 (i.e., average cluster with total expected count around 80). On the other hand, true relative risks of around 2 are detected with high probability as soon as the SF is 2 (which corresponds, on average, to a cluster with total expected count of 40).

The contrasting behavior of the MIX model is again apparent in Table 8 when one compares the results for the θ =1.5 scenario with the other columns. For Simu 1 and Simu 2 the sensitivity is generally below that of the BYM model and especially when the true relative risk is 1.5; single clusters with θ = 1.5 are simply not detected. In the 1% cluster case expected counts of at least 20 (10) are necessary to be over 95% certain of detecting a true relative risk of 2 (3) (Table 8). Note that the results of the last line of Table 8 should be discounted in view of the high probability of false-positive results corresponding to this scenario (Simu 3) for the *D*(0.05, 1.5) rule shown in Table 4. Thus, it is apparent that for the MIX model, it is hard to calibrate a good decision rule appropriate for a variety of spatial patterns of elevated risk. In Table 5 we summarize the results corresponding to the decision rule *D*(0.4, 1.5), which offers a reasonable compromise between keeping the rate of false positives below 7% and an acceptable detection rate of true clusters. With this rule true relative risks of 1.65 with an SF of 2 (i.e., average cluster with total expected count slightly under 40) or larger have more than a 50% chance of being detected, and true relative risks of around 2 are nearly always detected. However, this model does not detect a true relative risk as small as 1.3.

## Discussion

This comprehensive simulation study highlights some important points to be considered in interpreting any disease-mapping exercise based on hierarchical Bayesian procedures. First, the necessary control of false positives is indeed achieved using any of the models described. However, this is accompanied by a strong smoothing effect that renders the detection of localized increases in risk nearly impossible if these are not based on large (3-fold or more) excess risks or, in the case of more moderate (2-fold) excess risks, substantial expected counts of approximately 50 or more. Thus, in any study it is important to report the range of expected counts across the map and to calibrate any conclusions regarding the relative risks with respect to these expected counts.

In general Bayes procedures offer a tradeoff between bias and variance reduction of the estimates. Particularly in cases where the sample size is small, they produce a set of point estimates that have good properties in terms of minimizing squared error loss (Carlin and Louis 2000). This variance reduction is attained through borrowing information resulting from the adopted hierarchical structure, leading to Bayes point estimates shrunk toward a value related to the distribution of all the units included in the hierarchical structure. The effect of shrinkage is thus dependent on the prior structure that has been assumed and conditional on the latter being close to the true model in some sense. Consequently, different prior structures will lead to different shrinkage. Note that the desirable properties of the estimates thus obtained will depend on the ultimate goal of the estimation exercise. If producing a set of point estimates of the relative risk is the aim, then posterior means of the relative risk are best in squared error loss terms. However, if the goal is to estimate the histogram or the ranks of the area relative risks, different loss function should be considered. The desirability and difficulty of simultaneously achieving these triple goals has been discussed by Shen and Louis (1998) and has been illustrated in spatial case studies by Conlon and Louis (1999) and Stern and Cressie (1999). In our study, we focus on the goal of estimating the overall spatial pattern of risk, which involves producing and interpreting a set of point estimates that will not only give a good indication of the presence of heterogeneity in the relative risks but also highlight where on the map this heterogeneity arises and whether this is linked to isolated high- and/or low-risk areas or to more general spatial aggregation of areas of similar high or low risk. Inference about the latter will depend on the sensitivity and specificity of the posterior risk estimates, as discussed in this article. If the goal is purely the testing of heterogeneity, other methods could be used, such as the Potthoff-Whittinghill test or scan statistics [see Wakefield et al. (2000) for review] that test for particular prespecified patterns of overdispersion. Conversely, if the aim is a local study around a point source, then again, the disease-mapping framework is not appropriate, and focused models that make use of the additional information about the location of the putative cluster of high risk are required (Morris and Wakefield 2000).

We have shown that besides reporting and mapping the mean posterior relative risk, the whole posterior distribution can be usefully exploited to try to detect true raised-risk areas. For the BYM model, decision rules based on computing the probability that the relative risk is above 1 with a cutoff between 70 and 80% gives a specific rule. With this type of rule an average expected count of 20 in each of the raised-risk areas leads to a 50% chance of detecting a true relative risk of 1.5, but at least a 75% chance if the true relative risk is 2. For the same scenarios, the posterior mean relative risks are 1.05 and 1.23, respectively, showing that the posterior probabilities rather than the mean posterior relative risks are crucial for interpreting results from the BYM model. On the other hand, 3-fold increases in the relative risk are detected almost certainly with average expected counts of only 5 per area, although the mean of the posterior distribution is typically smoothed to about half the true excess. Note that the performance of the BYM model does improve when the risk is raised in a small group of contiguous areas with similar expected counts rather than in a single area because of the way spatial correlation is taken into account in these models.

We found no notable difference in performance between the BYM model, which uses a Gaussian distribution, and the L1 BYM version, which uses a heavier-tailed, double-exponential distribution. This finding is in agreement with that of an earlier simulation study (Best et al. 1999) that compared these two models. However, there were some clear differences between the BYM models and the spatial allocation model MIX. The performance of the latter model is characterized by an all-or-none feature in the sense that it tends to allocate the true raised-risk areas to either an elevated risk group or to a background group, depending on how much uncertainty is present in the data. If the information from the data is sufficient (i.e., moderate-size expected counts and/or high true excess risks) the MIX model is able to separate the raised-risk and background areas quite well, producing considerably less smoothing of the raised-risk estimates than BYM. When the information in the data is sparse, uncertainty in the groupings leads to more smoothing than the BYM. This type of dichotomy makes any decision rule exploiting the posterior distribution of the relative risks hard to calibrate and less useful than for the BYM model. The MIX model is best used for providing estimates of the underlying magnitude of the relative risks if those are clearly raised rather than as a tool for detecting the presence of areas with excess risk in a decision rule context.

## Conclusion

We have quantified to what extent some usual and some more recently developed Bayesian disease-mapping models are conservative, in the sense that they have low sensitivity for detecting raised-risk areas that have only a small excess risk but that, conversely, any identified patterns of elevated risk are, on the whole, specific. We would view this amount of conservatism as a positive feature, as we wish to avoid false alarms when investigating spatial variation in disease risk. However, the magnitude of the risk in any areas identified as raised is likely to be considerably underestimated, and it is worth investigating a range of spatial priors that produce different amounts of smoothing. Given that most environmental risks are small, it is clear that such methods are seriously underpowered to detect them. This represents a major limitation of the small-area disease-mapping approach, although exploiting the full posterior distribution of the relative risk estimates using the decision rules proposed here can improve the discrimination between areas with background and elevated rates. For localized excesses where the geographic source of the risk can be hypothesized, these methods are not appropriate, and focused tests should be used instead. Future applications of small-area disease-mapping methods should therefore consider carefully the tradeoff between size of the areas, size of the expected counts, and the anticipated magnitude and spatial structure of the putative risks. Recently proposed multivariate extensions of Bayesian disease-mapping models (e.g., Gelfand and Vounatsou 2003; Knorr-Held and Best 2001) also deserve further consideration, as they may lead to improved power by enabling risk estimates to borrow information across multiple diseases that share similar etiologies as well as across areas.

## Figures and Tables

**Table 1:** Posterior mean relative risk estimates for the raised-risk areas for the BYM model (average over replicate data sets).

	SF = 1			SF = 2			SF = 4			SF = 10		
Raised-risk area	θ = 1.5	θ = 2	θ = 3	θ = 1.5	θ = 2	θ = 3	θ = 1.5	θ = 2	θ = 3	θ = 1.5	θ = 2	θ = 3
Simu 1												
10% area (*E* = 0.84)	1.01	1.02	1.06	1.01	1.02	1.12	1.01	1.03	1.20	1.01	1.07	1.40
25% area (*E* = 1.10)	1.03	1.04	1.10	1.00	1.03	1.15	1.01	1.05	1.28	1.02	1.09	1.52
50% area (*E* = 1.92)	1.02	1.05	1.15	1.00	1.05	1.28	1.02	1.08	1.46	1.03	1.16	1.79
75% area (*E* = 5.37)	1.03	1.05	1.31	1.03	1.07	1.55	1.04	1.12	1.86	1.05	1.33	2.35
90% area (*E* = 7.38)	1.03	1.07	1.34	1.03	1.10	1.62	1.04	1.15	2.07	1.07	1.40	2.47
Simu 2												
1% cluster (&*Emacr;* = 5.42)	1.04	1.08	1.45	1.04	1.14	1.76	1.05	1.23	2.11	1.09	1.45	2.43
Simu 3	θ* = 1.35	θ* = 1.65	θ* = 2.1	θ* = 1.35	θ* = 1.65	θ* = 2.1	θ* = 1.35	θ* = 1.65	θ* = 2.1	θ* = 1.35	θ* = 1.65	θ* = 2.1
20 × 1% clusters (*Ē* range: 0.77–11.6)	1.04	1.23	1.63	1.07	1.3	1.74	1.12	1.38	1.84	1.19	1.48	1.95

**Table 2:** Posterior mean relative risk estimates for the raised-risk areas for the L1-BYM model (average over replicate data sets).

	SF = 1			SF = 2			SF = 4			SF = 10		
Raised-risk area	θ = 1.5	θ = 2	θ = 3	θ = 1.5	θ = 2	θ = 3	θ = 1.5	θ = 2	θ = 3	θ = 1.5	θ = 2	θ = 3
Simu 1												
10% area (*E* = 0.84)	1.01	1.02	1.05	1.01	1.02	1.12	1.01	1.02	1.16	1.01	1.07	1.21
25% area (*E* = 1.10)	1.01	1.03	1.11	1.00	1.04	1.15	1.00	1.06	1.24	1.03	1.09	1.35
50% area (*E* = 1.92)	1.01	1.03	1.16	1.00	1.05	1.28	1.01	1.08	1.55	1.03	1.17	2.22
75% area (*E* = 5.37)	1.02	1.05	1.32	1.03	1.08	1.56	1.03	1.13	1.98	1.05	1.35	2.67
90% area (*E* = 7.38)	1.04	1.07	1.48	1.03	1.13	1.93	1.05	1.25	2.43	1.08	1.60	2.72
Simu 2												
1% cluster (*Ē* = 5.42)	1.04	1.08	1.45	1.04	1.14	1.76	1.05	1.23	2.11	1.09	1.45	2.43
Simu 3	θ* = 1.35	θ* = 1.65	θ* = 2.1	θ* = 1.35	θ* = 1.65	θ* = 2.1	θ* = 1.35	θ* = 1.65	θ* = 2.1	θ* = 1.35	θ* = 1.65	θ* = 2.1
20 × 1% clusters (&*Emacr;* range: 0.77–11.6)	1.04	1.22	1.61	1.07	1.29	1.74	1.12	1.38	1.85	1.19	1.49	1.97

**Table 3:** Posterior mean relative risk estimates for the raised-risk areas for the MIX model (average over replicate data sets).

	SF = 1			SF = 2			SF = 4			SF = 10		
Raised-risk area	θ = 1.5	θ = 2	θ = 3	θ = 1.5	θ = 2	θ = 3	θ = 1.5	θ = 2	θ = 3	θ = 1.5	θ = 2	θ = 3
Simu 1												
10% area (*E* = 0.84)	1.00	1.01	1.02	1.00	1.02	1.27	1.00	1.01	1.53	1.01	1.10	2.50
25% area (*E* = 1.10)	1.00	1.02	1.09	1.00	1.01	1.17	1.00	1.05	1.80	1.01	1.22	2.67
50% area (*E* = 1.92)	1.00	1.02	1.25	1.00	1.04	1.88	1.00	1.23	2.78	1.02	1.72	3.02
75% area (*E* = 5.37)	1.00	1.03	1.57	1.00	1.07	2.44	1.01	1.42	2.91	1.04	1.87	3.02
90% area (*E* = 7.38)	1.00	1.03	1.60	1.01	1.09	2.46	1.01	1.49	2.91	1.06	1.89	3.02
Simu 2												
1% cluster (&*Emacr;* = 5.42)	1.02	1.06	1.98	1.01	1.25	2.66	1.03	1.72	2.92	1.21	1.92	2.98
Simu 3	θ* = 1.35	θ* = 1.65	θ* = 2.1	θ* = 1.35	θ* = 1.65	θ* = 2.1	θ* = 1.35	θ* = 1.65	θ* = 2.1	θ* = 1.35	θ* = 1.65	θ* = 2.1
20 × 1% clusters (&*Emacr;* range: 0.77–11.6)	1.02	1.19	1.55	1.05	1.31	1.64	1.12	1.44	1.81	1.31	1.55	2.06

**Table 4:** False-positive rates (1 *–* specificity) for the three models.*^a^*

	SF = 1			SF = 2			SF = 4			SF = 10		
Background	θ = 1.5	θ = 2	θ = 3	θ = 1.5	θ = 2	θ = 3	θ = 1.5	θ = 2	θ = 3	θ = 1.5	θ = 2	θ = 3
BYM												
Simu 1	0.08	0.10	0.05	0.04	0.06	0.04	0.03	0.08	0.06	0.03	0.05	0.08
Simu 2	0.07	0.06	0.06	0.05	0.05	0.06	0.05	0.05	0.07	0.04	0.08	0.10
Simu 3*^b^*	0.02	0.03	0.02	0.02	0.03	0.02	0.03	0.03	0.01	0.03	0.02	0.01
L1-BYM												
Simu 1	0.05	0.09	0.06	0.06	0.10	0.05	0.03	0.06	0.06	0.05	0.05	0.08
Simu 2	0.07	0.09	0.06	0.05	0.07	0.06	0.05	0.06	0.06	0.04	0.07	0.08
Simu 3*^b^*	0.04	0.03	0.02	0.02	0.03	0.02	0.03	0.03	0.02	0.03	0.02	0.01
MIX												
Simu 1	0.00	0.04	0.00	0.01	0.04	0.00	0.03	0.02	0.00	0.02	0.00	0.08
Simu 2	0.00	0.01	0.11	0.00	0.04	0.04	0.00	0.06	0.01	0.01	0.02	0.00
Simu 3*^b^*	0.02	0.51	0.44	0.02	0.52	0.25	0.01	0.33	0.12	0.00	0.14	0.03

**a**Decision rules are *D*(0.8, 1) for BYM and L1-BYM and *D*(0.05, 1.5) for MIX.

**b**For Simu 3, θ* = 1.35, 1.65, or 2.1 instead of θ = 1.5, 2, or 3, respectively.

**Table 5:** Simu 3: performance of the BYM and MIX models under alternative decision rules.

	SF = 1			SF = 2			SF = 4			SF = 10		
	θ* = 1.35	θ* = 1.65	θ* = 2.1	θ* = 1.35	θ* = 1.65	θ* = 2.1	θ* = 1.35	θ* = 1.65	θ* = 2.1	θ* = 1.35	θ* = 1.65	θ* = 2.1
BYM – *D*(0.7, 1)												
Probability (false detection)	0.10	0.07	0.05	0.07	0.07	0.04	0.08	0.06	0.03	0.08	0.05	0.02
Probability (true detection)	0.23	0.51	0.71	0.36	0.68	0.84	0.56	0.82	0.93	0.81	0.95	0.99
MIX – *D*(0.4,1.5)												
Probability (false detection)	0.00	0.03	0.07	0.00	0.06	0.05	0.00	0.07	0.03	0.00	0.03	0.01
Probability (true detection)	0.00	0.23	0.76	0.00	0.62	0.88	0.00	0.84	0.93	0.00	0.93	0.98

**Table 6:** Sensitivity (1 *–* false-negative rate) for the BYM model.*^a^*

	SF = 1			SF = 2			SF = 4			SF = 10		
Raised-risk area	θ = 1.5	θ = 2	θ = 3	θ = 1.5	θ = 2	θ = 3	θ = 1.5	θ = 2	θ = 3	θ = 1.5	θ = 2	θ = 3
Simu 1												
10% area (*E* = 0.84)	0.08	0.06	0.08	0.04	0.02	0.36	0	0.06	0.68	0.02	0.42	0.98
25% area (*E* = 1.10)	0.36	0.48	0.38	0.20	0.24	0.36	0.20	0.50	0.82	0.28	0.54	1
50% area (*E* = 1.92)	0.32	0.48	0.40	0.16	0.32	0.66	0.24	0.66	0.98	0.30	0.96	1
75% area (*E* = 5.37)	0.08	0.30	0.74	0.12	0.52	0.98	0.22	0.76	1	0.66	1	1
90% area (*E* = 7.38)	0.12	0.22	0.74	0.10	0.64	0.98	0.34	0.88	1	0.88	1	1
Simu 2												
1% cluster (*Ē* = 5.42)	0.18	0.42	0.95	0.30	0.74	1	0.53	0.97	1	0.90	1	1
Simu 3	θ* = 1.35	θ* = 1.65	θ* = 2.1	θ* = 1.35	θ* = 1.65	θ* = 2.1	θ* = 1.35	θ* = 1.65	θ* = 2.1	θ* = 1.35	θ* = 1.65	θ* = 2.1
20 × 1% clusters (*Ē* range: 0.77–11.6)	0.09	0.34	0.56	0.17	0.51	0.74	0.37	0.71	0.88	0.66	0.90	0.94

**a**Decision rule is *D*(0.8, 1).

**Table 7:** Probability of true detection (sensitivity) for the L1-BYM model.*^a^*

	SF = 1			SF = 2			SF = 4			SF = 10		
Raised-risk area	θ= 1.5	θ= 2	θ= 3	θ= 1.5	θ= 2	θ= 3	θ= 1.5	θ= 2	θ= 3	θ= 1.5	θ= 2	θ= 3
Simu 1												
10% area (*E* = 0.84)	0.02	0.04	0.04	0.04	0.08	0.32	0.02	0.02	0.54	0.04	0.28	0.54
25% area (*E* = 1.10)	0.26	0.34	0.38	0.24	0.38	0.40	0.16	0.46	0.88	0.44	0.52	0.98
50% area (*E* = 1.92)	0.28	0.38	0.42	0.30	0.42	0.66	0.26	0.56	0.96	0.44	0.86	1
75% area (*E* = 5.37)	0.08	0.24	0.74	0.06	0.50	0.94	0.20	0.78	1	0.68	1	1
90% area (*E* = 7.38)	0.16	0.22	0.76	0.10	0.68	0.98	0.24	0.90	1	0.86	1	1
Simu 2												
1% cluster (*Ē* = 5.42)	0.17	0.35	0.91	0.23	0.64	1	0.39	0.95	1	0.85	1	1
Simu 3	θ* = 1.35	θ* = 1.65	θ* = 2.1	θ* = 1.35	θ* = 1.65	θ* = 2.1	θ* = 1.35	θ* = 1.65	θ* = 2.1	θ* = 1.35	θ* = 1.65	θ* = 2.1
20 × 1% clusters (*Ē* range: 0.77–11.6)	0.10	0.31	0.55	0.16	0.48	0.75	0.35	0.70	0.89	0.65	0.90	0.98

**a**Decision rule is *D*(0.8, 1).

**Table 8:** Probability of true detection (sensitivity) for the MIX model.*^a^*

	SF = 1			SF = 2			SF = 4			SF = 10		
Raised-risk area	θ= 1.5	θ= 2	θ= 3	θ= 1.5	θ= 2	θ= 3	θ= 1.5	θ= 2	θ= 3	θ= 1.5	θ= 2	θ= 3
Simu 1												
10% area (*E* = 0.84)	0	0	0.05	0	0.02	0.35	0	0.04	0.56	0	0.31	0.54
25% area (*E* = 1.10)	0	0.02	0.20	0	0.01	0.30	0	0.16	0.72	0.06	0.53	0.98
50% area (*E* = 1.92)	0	0.02	0.33	0	0.10	0.77	0	0.51	0.98	0.05	0.94	1
75% area (*E* = 5.37)	0	0.02	0.51	0	0.18	0.90	0	0.67	0.99	0.10	0.98	1
90% area (*E* = 7.38)	0	0.05	0.55	0	0.19	0.93	0	0.68	0.99	0.14	0.98	1
Simu 2												
1% cluster (*Ē* = 5.42)	0.02	0.10	0.86	0.01	0.46	0.99	0.05	0.95	1	0.47	1.00	1.00
Simu 3	θ* = 1.35	θ* = 1.65	θ* = 2.1	θ* = 1.35	θ* = 1.65	θ* = 2.1	θ* = 1.35	θ* = 1.65	θ* = 2.1	θ* = 1.35	θ* = 1.65	θ* = 2.1
20 × 1% clusters (*Ē* range: 0.77–11.6)	0.04	0.85	0.99	0.04	0.99	0.99	0.06	0.99	0.99	0.0	0.99	1.00

**a**Decision rule is *D*(0.5, 1.5).

**Figure B1:**
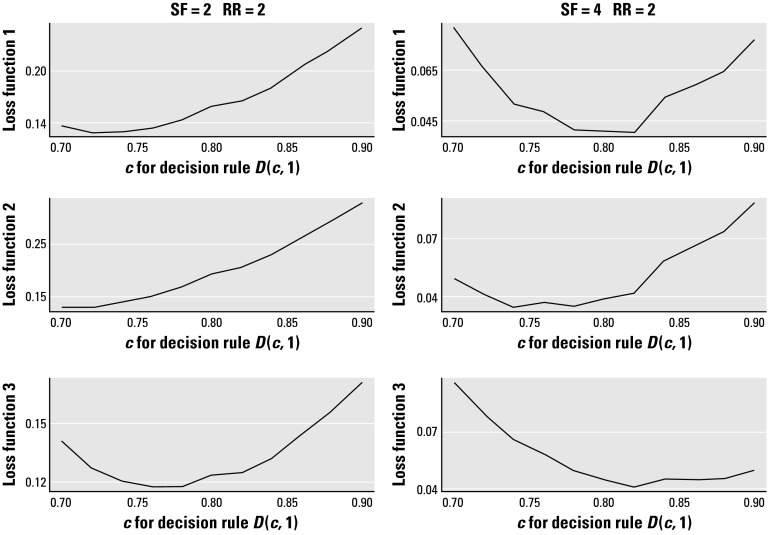
Variation of the total error rate (loss function) as function of the cutoff probability *c* for different weighting of the two types of errors (false positive and false negative). Results shown are for the Simu 2 using the BYM model.

## Appendix A

### Generation of the observed cases for Simu 3 using the multinomial distribution.

For Simu 3, the number of cases for each of the 532 areas is generated using the multinomial distribution as follows:

where *N* is the total number of cases in the study region and is set equal (to the nearest integer) to the sum of the expected counts across all 532 areas. Hence *N* = 1,732 for the SF = 1 scenario and appropriate multiples of this for the other SFs.

The parameter θ*_i_* represents the relative risk in area *i* relative to some nominal external reference rate. However, the constraint Σ*_i_* *Y**_i_* *= N =* Σ*_i_* *E**_i_* imposed by the multinomial sampling effectively rescales the true relative risk in each area to be

The interpretation of θ*^*^* *_i_* is the relative risk in area *i* relative to the average risk in the study region.

## Appendix B

### Tradeoff between false-positive and false-negative rates for different decision rules.

Figure B1 shows three different loss functions representing weighted tradeoffs between the two types of errors: false positive and false negative, associated with the *D(c*, 1) decision rule for detecting raised-risk areas using the BYM model, plotted against cutoff *c*. Defining as in the text the false-negative rate to be the probability of failing to detect a true raised risk (i.e., 1 *–* sensitivity), and the false-positive rate to be the probability of false detection of a background area as corresponding to a raised risk (i.e., 1 *–* specificity), the three loss functions used are as follows:

with each error being equally weighted.

where we weight the false negative error as twice as bad as the lack of specificity.

where we weight the lack of specificity as twice as bad as the false negative.

We wish to choose *c* to minimize the losses, and the graphs show that, on average, a value of around 0.7–0.8 is appropriate. Note that the plots in Figure B1 are based on Simu 2 with SF = 2 or 4. For a small number of other scenarios (mainly with SF = 1), a value of *c* < 0.7 was needed to minimize the loss. However, for consistency, we have used the same value of *c* (= 0.8) for all the BYM and L1-BYM results presented in this article.
